# Association between smoking behavior and sleep health among South Korean adolescents: A cross-sectional study 2020–2023

**DOI:** 10.18332/tid/211247

**Published:** 2025-12-31

**Authors:** Nagyeong Cho, Sung-il Cho

**Affiliations:** 1 Department of Public Health Science, Graduate School of Public Health, Seoul National University, Seoul, Republic of Korea; 2 Institute of Health and Environment, Graduate School of Public Health, Seoul National University, Seoul, Republic of Korea

**Keywords:** adolescents, tobacco use, smoking behavior, sleep health, poly use

## Abstract

**INTRODUCTION:**

Tobacco use has been associated with poor sleep quality among adolescents. Given the rising prevalence of poly tobacco use among South Korean adolescents, it is crucial to examine its cumulative impact on sleep health. This study aims to assess the association between various smoking behaviors and sleep health outcomes in this population.

**METHODS:**

This cross-sectional pooled secondary data analysis utilized data from the 2020–2023 Korea Youth Risk Behavior Web-based Survey (KYRBS), comprising 172457 middle and high school students (aged 12–18 years). Information was collected via a self-administered web-based questionnaire. Multinomial logistic regression models were employed to evaluate the relationship between smoking behaviors with sleep health, adjusting for potential confounders.

**RESULTS:**

Overall, 40.3% of tobacco users and 25.3% of non-users reported insufficient sleep accompanied by poor satisfaction. Adolescents engaged in triple use (concurrent use of conventional cigarettes, e-cigarettes, and heated tobacco products) demonstrated the highest odds of insufficient and poor sleep (adjusted odds ratio, AOR=2.32; 95% confidence interval CI: 2.01–2.67). A graded pattern was observed whereby increased poly tobacco use corresponded to poorer sleep outcomes.

**CONCLUSIONS:**

Poly tobacco use is significantly associated with poor sleep health among South Korean adolescents. Future longitudinal studies are needed to establish causal pathways and provide sufficient evidence to guide effective interventions and policies.

## INTRODUCTION

Sleep health is a multidimensional concept encompassing duration, quality, timing, regularity, and the absence of disturbances^1^. Adequate sleep promotes physical and mental health, whereas sleep disruptions are associated with adverse health outcomes such as metabolic disorders, impaired cognitive function, and increased vulnerability to chronic conditions^2^. Poor sleep health has become a pervasive issue globally which is exacerbated by modern social trends such as extended screen time, high levels of stress, and changing lifestyle behaviors^3^.

Adolescents are at a developmental stage where sleep plays a pivotal role in their physical, cognitive, and emotional growth. For optimal health and functioning, it is recommended that adolescents obtain 8 to 10 hours of sleep per night^4^. South Korean adolescents average only 6.2 hours of sleep, and 76.0% of adolescents experienced poor sleep satisfaction in 2023^5^. Contributing socioenvironmental and behavioral factors include late-night tutoring in a highly competitive academic environment and excessive screen time^6^. These factors drive sleep deprivation and irregular schedules, which lower sleep quality^7-9^. Poor sleep also undermines physical and mental well-being affecting academic performance and social functioning^10^.

Adolescents can be categorized into those who attend school and those who do not. School-attending adolescents face distinct challenges to sleep health. Fixed schedules of schools, extracurricular activities, and intensive academic workloads often delay bedtimes and reduce total sleep duration due to early school start times^11^. Additionally, behavioral factors, specifically excessive caffeine intake and prolonged use of electronic devices, and increased sedentary times, enhance disruptions in sleep patterns^12^. The interplay between these factors creates persistent barriers to healthy sleep.

Tobacco use is another key factor. Nicotine in tobacco products acts as a stimulant that reduces total sleep time and increases sleep latency, impairing sleep quality^13^. Research suggests adolescents who smoke are more likely to report insufficient and poor sleep than non-smokers^14^. With the growing availability of alternative tobacco products worldwide, the sale of electronic cigarettes (ECs) and heated tobacco products (HTPs) have expanded the tobacco market in South Korea and gained popularity among adolescents^15^. Likewise, the rising prevalence of multiple or poly tobacco use among this demographic may further complicate the association between tobacco consumption and sleep health^16^.

School-attending adolescents who use tobacco may be especially vulnerable to the compounded effects of tobacco use on sleep health. Academic demands combined with the physiological and psychological effects of nicotine are likely to increase the likelihood of poor sleep^17-19^. Nicotine dependence in adolescence results in shorter sleep, lower satisfaction, and greater daytime sleepiness, which could lead to a decrease in their ability to perform academically and socially^20^. Understanding how tobacco use behaviors influence the sleep health of school-attending adolescents in the domestic context is important to promote both better sleep health and smoking cessation.

This study seeks to address the gap in the literature by examining the association between smoking behavior and sleep health among South Korean adolescents. By analyzing patterns of tobacco use and their relationship with sleep duration and satisfaction, the findings aim to clarify these relationships and inform future research on adolescent sleep health.

## METHODS

### Study design and data source

This study is a cross-sectional pooled secondary data analysis based on the Korea Youth Risk Behavior Web-based Survey (KYRBS). We analyzed data collected from 2020 to 2023. The KYRBS is an annual, nationally representative survey of middle and high school students in South Korea, conducted by the Korea Disease Control and Prevention Agency (KDCA). The survey uses a self-administered, anonymous, web-based questionnaire to collect information on health behaviors across various domains including smoking, alcohol consumption, and sleep health.

### Study population

**Figure 1:**
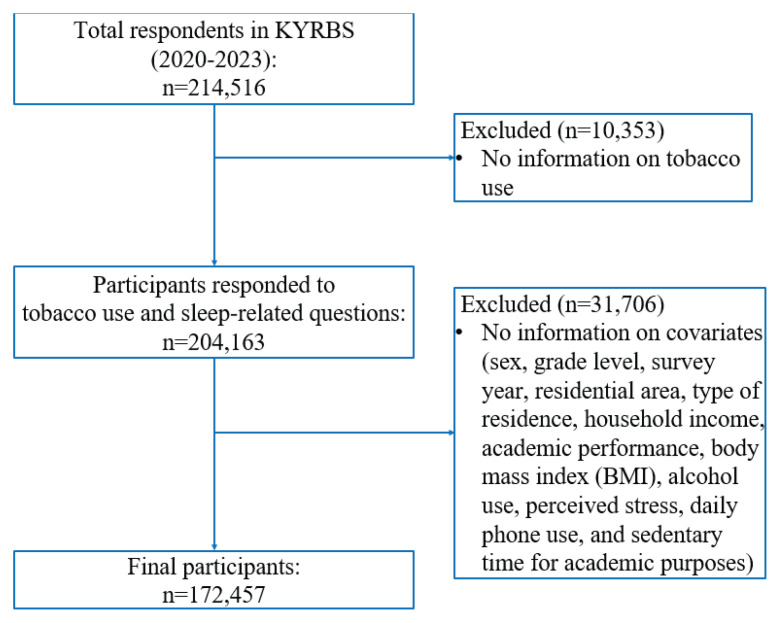
Flow chart of the study population, South Korea, Korea Youth Risk Behavior Web-based Survey (KYRBS), 2020–2023

The initial dataset included 214526 adolescents. Participants were eligible if they were middle or high school students aged 12–18 years and had completed responses for tobacco use and sleep health variables. Adolescents with missing values in key variables (n=42059) were excluded, leaving a final analytic sample of 172457 adolescent (Figure 1).

### Measures

*Sleep health*

The primary outcome was sleep health assessed using two questionnaire-based measures: sleep duration and sleep satisfaction. Sleep duration was calculated from self-reported bedtime and wake-up time during weekdays. Following national survey standards and prior reports that South Korean adolescents average 6.2 hours of sleep per night in 2023, we categorized sleep as sufficient (>6 hours) or insufficient (≤6 hours)^5^. Sleep satisfaction was measured by self-reported adequacy of sleep for recovery. Responses of ‘very satisfied’, ‘satisfied’, or ‘neutral’ were grouped as good, while ‘dissatisfied’ or ‘very dissatisfied’ were grouped as poor. These two dimensions were cross-analyzed to create four categories: sufficient and good, sufficient and poor, insufficient and good, and insufficient and poor. This combined measure allowed for a more comprehensive assessment of both quantitative and subjective sleep health.

*Type of tobacco use*

Tobacco use in the past 30 days was classified into six categories: non-use (never used any tobacco product), former use (previous use but none in the past 30 days), current exclusive conventional cigarette (CC) use, current exclusive non-combustible nicotine product (NCNP) use (EC or HTP), current dual use (any two product types), and current triple use (concurrent use of CC, EC, and HTP).

*Tobacco use behavior*

Tobacco use behavior was further classified by frequency in the past 30 days: former use (no use), non-daily use (<30 days), and daily use (30 days). For poly users, intensity patterns were also considered: light poly use (non-daily CC and NCNP), predominant smoking (daily CC with non-daily NCNP), and heavy poly use (daily use of both CC and NCNP). These categories allowed for a clearer assessment of both single- and multiple-product use patterns^21^.

### Covariates

The covariates were selected based on prior literature linking then to tobacco use and sleep outcomes^22^. These were grouped into sociodemographic and health-related factors. Sociodemographic factors are sex (male, female), grade level (7th-12th), survey year (2020–2023), residence (rural, small/middle-sized city, metropolitan), type of residence (living with family, dormitory, or other arrangement), household income (self-reported on a five-point Likert scale and recoded into low, middle, and high), and academic performance (self-reported school grades in the past 12 months, categorized into low, middle, and high).

Health-related behaviors included several self-reported measures. Body mass index (BMI, kg/m^2^) was calculated from self-reported height and weight, categorized into underweight (<18.5), normal (18.5–24.9), overweight (25.0–29.9), and obese (≥30.0). Alcohol use was assessed as a binary variable, distinguishing between adolescents who had ever used alcohol and those who had never used it. Perceived stress was measured and classified into two categories: high stress and below high stress. Daily phone use was grouped into four categories based on the average number of hours spent per day: <2, 2–4, 4–6 and ≥6 hours. Sedentary time for academic purposes was also measured and classified into three categories: <7, 7–11, and ≥11 hours per day. This measure reflected time spent in school, private academies, or self-study, which aligns with the typical schedules of South Korean adolescents.

### Statistical analysis

All analyses were conducted using SAS 9.4 (SAS Institute, Cary, NC). The complex survey design of the KYRBS was taken into account by applying sampling weights, stratification, and clustering to yield nationally representative estimates. Descriptive analyses were first performed to summarize the distribution of demographic, behavioral, and tobacco use characteristics. Frequencies and percentages were calculated, and these were further stratified by the four categories of sleep health. Chi-squared tests were applied to examine the unadjusted associations between tobacco use categories and sleep health.

Multinomial logistic regression models were fitted to estimate adjusted odds ratios (AORs) with 95% confidence intervals (CIs) for the association between tobacco use patterns and the four-category sleep health outcome. Non-users of tobacco products served as the reference group. Interaction analyses were conducted to examine whether the association between tobacco use and sleep health varied by subgroups. To simplify interpretation and ensure sufficient sample sizes within subgroups, sleep health categories were dichotomized into good versus poor sleep. Binomial logistic regression was applied for these models, while the primary analyses used multinomial logistic regression. Detailed results are provided in the Supplementary file. All statistical tests were two-tailed with statistical significance defined as p<0.05.

## RESULTS

### Study population and sleep health distribution

A total of 172457 adolescents aged 12–18 years were included in the analysis. Table 1 presents the distribution of sleep health across the study population. Overall, 39.6% reported sufficient sleep with good satisfaction, while 26.1% experienced insufficient sleep with poor satisfaction. The remaining participants were distributed between sufficient but poor sleep satisfaction (14.5%) and insufficient sleep with good satisfaction (19.8%).

**Table 1:** Sleep health of adolescents in South Korea, by tobacco use status, Korea Youth Risk Behavior Web-based Survey 2020–2023 (N=172457)

*Variables*	*Sleep Health*			
	*Sufficient and Good*	*Sufficient and Poor*	*Insufficient and Good*	*Insufficient and Poor*
	*n (%)*	*n (%)*	*n (%)*	*n (%)*
**Total**	68321 (39.6)	24970 (14.5)	34111 (19.8)	45055 (26.1)
**Smoking status**				
Non-user	66091 (40.6)	23659 (14.5)	31861 (19.6)	41138 (25.3)
User	2230 (23.0)	1311 (13.5)	2250 (23.2)	3917 (40.3)
Former user	324 (26.2)	207 (16.8)	265 (21.5)	439 (35.6)
**Type of tobacco product use**				
Current exclusive CC use	859 (25.3)	417 (12.3)	804 (23.6)	1,321 (38.8)
Current exclusive NCNP use	210 (25.5)	115 (14.0)	193 (23.5)	305 (37.1)
Current dual use^a^	526 (21.0)	354 (14.2)	569 (22.7)	1,053 (42.1)
Current triple use^b^	311 (17.8)	218 (12.5)	419 (24.0)	799 (45.7)

P-values from Pearson chi-squared tests incorporating complex survey weights, strata, and clusters. Among each category, all variables showed statistically significant differences by sleep health status (p<0.0001). CC: conventional cigarette. NCNP: non-combustible nicotine product, including electronic cigarettes (ECs) and heated tobacco products (HTPs).

a Current dual use: concurrent use of any two products (e.g. CC + EC, CC + HTP, or EC + HTP).

b Current triple use: concurrent use of all three products (CC, EC, and HTP).

When stratified by tobacco use, 40.6% of non-users reported sufficient and good sleep, while only 23.0% of tobacco users reported sufficient and good sleep. Sleep health outcomes worsened progressively across categories of tobacco use: 35.6% of former users, 38.8% of exclusive conventional cigarette (CC) users, 37.1% of exclusive non-combustible nicotine product (NCNP) users, 42.1% of dual users, and 45.7% of triple users experienced insufficient sleep with poor satisfaction (p<0.0001).

### Sociodemographic characteristics and sleep health

Table 2 shows the distribution of sleep health by sociodemographic factors. Male participants more frequently reported sufficient and good sleep (46.4%) compared to females (32.7%), while females reported a higher likelihood of insufficient and poor sleep (32.6% vs 19.8%). Sleep health declined consistently with grade level: 57.2% of 7th grade students reported sufficient and good sleep, compared to 26.2% of 12th grade students. Similarly, insufficient and poor sleep increased from 12.3% for 7th grade to 36.9% for 12th grade students (p<0.0001). Academic performance and sedentary time also showed significant associations. Students with high academic performance reported the best sleep outcomes, whereas those with ≥11 hours of sedentary time per day had the highest proportion of insufficient and poor sleep.

**Table 2:** Characteristics of adolescents in South Korea, by sleep health, Korea Youth Risk Behavior Web-based Survey 2020–2023 (N=172457)

*Variables*	*Categories*	*Sleep health*			
		*Sufficient and good*	*Insufficient and poor*	*Sufficient and good*	*Insufficient and poor*
		*n (%)*	*n (%)*	*n (%)*	*n (%)*
**Sex**	Male	40417 (46.4)	12643 (14.5)	16794 (19.3)	17269 (19.8)
	Female	27904 (32.7)	12327 (14.5)	17317 (20.3)	27786 (32.6)
**Grade**	7	19287 (57.2)	5941 (17.6)	4346 (12.9)	4150 (12.3)
	8	15710 (48.8)	5548 (17.3)	4933 (15.3)	5973 (18.6)
	9	13020 (42.3)	5147 (16.7)	5445 (17.7)	7179 (23.3)
	10	7730 (28.2)	3306 (12.1)	6486 (23.7)	9862 (36.0)
	11	6605 (25.8)	2749 (10.7)	6778 (26.5)	9481 (37.0)
	12	5969 (26.2)	2279 (10.0)	6123 (26.9)	8410 (36.9)
**Residence**	Rural	5526 (42.5)	2198 (16.9)	2388 (18.4)	2877 (22.2)
	Small to middle-sized city	34407 (40.7)	12931 (15.3)	15894 (18.8)	21263 (25.2)
	Metropolitan city	28388 (37.9)	9841 (13.1)	15829 (21.1)	20915 (27.9)
**Household income level**	High	30208 (42.8)	9707 (13.7)	13594 (19.2)	17160 (24.3)
	Moderate	31818 (38.7)	11,945 (14.5)	16,705 (20.3)	21,680 (26.4)
	Low	6295 (32.1)	3318 (16.9)	3812 (19.4)	6215 (31.6)
**Type of residence**	Living with family	66240 (40.0)	23846 (14.4)	32754 (19.8)	42765 (25.8)
	Dormitory	1471 (29.2)	878 (17.4)	959 (19.1)	1726 (34.3)
	Other	610 (33.6)	246 (13.5)	398 (21.9)	564 (31.0)
**Academic performance**	High	28062 (40.6)	9976 (15.2)	11003 (16.7)	16810 (25.5)
	Moderate	21338 (40.6)	7066 (13.4)	11,063 (21.0)	13,117 (24.9)
	Low	18921 (35.0)	7928 (14.7)	12045 (22.3)	15128 (28.0)
**BMI**	Obese	47001 (39.4)	17,489 (14.6)	23236 (19.5)	31715 (26.6)
	Overweight	8959 (40.6)	3017 (13.7)	4702 (21.3)	5375 (24.4)
	Normal	6912 (41.1)	2414 (14.3)	3368 (20.0)	4143 (24.6)
	Underweight	5449 (38.6)	2050 (14.5)	2805 (19.9)	3822 (27.1)
**Alcohol use**	No	52061 (43.4)	17389 (14.5)	22726 (19.0)	27744 (23.1)
	Yes	16260 (31.0)	7581 (14.4)	11385 (21.7)	17311 (33.0)
**Perceived stress**	High	51241 (47.4)	13255 (12.3)	23338 (21.6)	20368 (18.8)
	Below high	17080 (26.6)	11715 (18.2)	10773 (16.8)	24687 (38.4)
**Hours of phone use per day**	>6	13464 (30.8)	5936 (13.6)	10148 (23.2)	14237 (32.5)
	4–6	19467 (37.7)	7592 (14.7)	10712 (20.7)	13887 (26.9)
	2–4	27134 (44.9)	9017 (14.9)	10677 (17.7)	13616 (22.5)
	<2	8256 (49.8)	2425 (14.6)	2574 (15.5)	3315 (20.0)
**Sedentary time** (hours for academic purposes)	<7	34149 (44.6)	10,687 (14.0)	15950 (20.8)	15815 (20.7)
	7–11	26924 (39.8)	10612 (15.7)	12496 (18.5)	17582 (26.0)
	>11	7248 (25.7)	3671 (13.0)	5665 (20.1)	11658 (41.3)
**Survey year**	2020	17868 (40.7)	4855 (11.1)	10,800 (24.6)	10,398 (23.7)
	2021	16825 (38.3)	7110 (16.2)	8030 (18.3)	11926 (27.2)
	2022	15301 (36.8)	6626 (16.0)	7562 (18.2)	12057 (29.0)
	2023	18327 (42.5)	6379 (14.8)	7719 (17.9)	10674 (24.8)

P-values from Pearson chi-squared tests incorporating complex survey weights, strata, and clusters. Across all categories, differences in sleep health were statistically significant (p<0.0001). BMI: body mass index (kg/m²), categorized as: underweight (<18.5), normal (18.5–24.9), overweight (25.0–29.9), and obese (≥30.0).

### Association between tobacco use and sleep health

**Figure 2:**
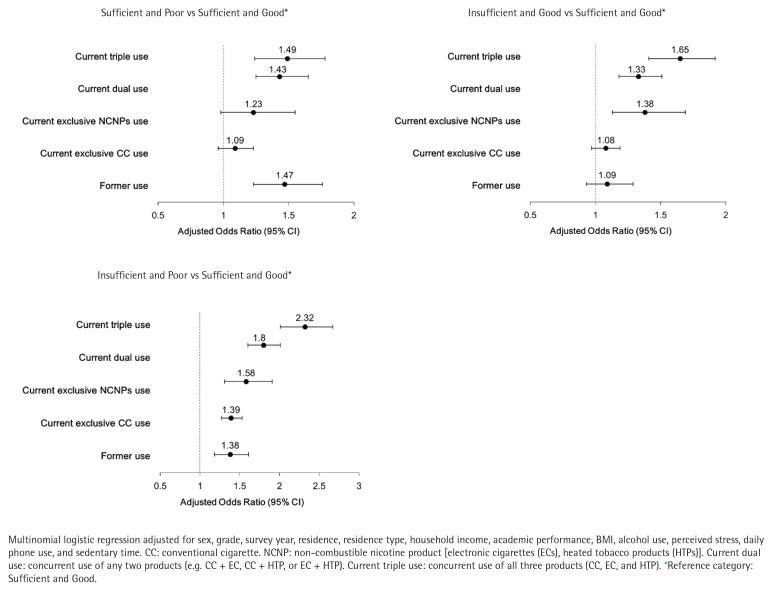
Forest plots of adjusted odds ratios (AOR, 95% CI) for sleep health by type of tobacco use, South Korea, Korea Youth Risk Behavior Web-based Survey (KYRBS), 2020–2023

Multinomial logistic regression results, adjusted for all covariates, are presented in Table 3. In these models, non-users served as the reference category for the independent variable, and sufficient and good sleep served as the reference category for the dependent variable. Compared to non-users, former users had higher odds of reporting insufficient and poor sleep (AOR=1.38; 95% CI: 1.18–1.61). Current exclusive CC users (AOR=1.39; 95% CI: 1.27–1.53) and exclusive NCNP users (AOR=1.58; 95% CI: 1.34–1.86) also demonstrated increased odds. Dual users showed even higher odds (AOR=1.80; 95% CI: 1.60–2.01), while triple users had the greatest likelihood of insufficient and poor sleep (AOR=2.32; 95% CI: 2.01–2.67) (Figure 2).

**Table 3:** Multinomial logistic regression, including all covariates, for associations between type of tobacco use and sleep health among adolescents, Korea Youth Risk Behavior Web-based Survey 2020–2023 (N=172457)

*Type of tobacco use (ref: non-use)*	*Sleep health* *(ref: Sufficient and good)*		
	*Sufficient and poor*	*Insufficient and good*	*Insufficient and poor*
	*AOR (95% CI)*	*AOR (95% CI)*	*AOR (95% CI)*
Former use	1.47 (1.23–1.76)***	1.09 (0.93–1.29)	1.38 (1.18–1.61)***
Current exclusive CC use	1.09 (0.96–1.23)	1.08 (0.97–1.19)	1.39 (1.27–1.53)***
Current exclusive NCNP use	1.23 (0.98–1.55)	1.38 (1.13–1.69)*	1.58 (1.31–1.91)***
Current dual use^a^	1.43 (1.25–1.65)***	1.33 (1.18–1.51)***	1.80 (1.60–2.01)***
Current triple use^b^	1.49 (1.24–1.78)***	1.65 (1.41–1.92)***	2.32 (2.01–2.67)***

Models adjusted for sex, grade, survey year, residence, type of residence, household income, academic performance, body mass index, alcohol use, perceived stress, daily phone use, and sedentary time for academic purposes. AOR: adjusted odds ratio. CC: conventional cigarette. NCNP: non-combustible nicotine product, including electronic cigarettes (ECs) and heated tobacco products (HTPs).

a Current dual use: concurrent use of any two products (e.g. CC + EC, CC + HTP, or EC + HTP).

b Current triple use: concurrent use of all three products (CC, EC, and HTP).

* p<0.05;

** p<0.001;

*** p<0.0001.

Analyses (Supplementary file Appendix A) further highlighted subgroup differences. Female adolescents had greater odds of poor sleep health compared to males (AOR=2.09; 95% CI: 2.03–2.15). Low academic performance was associated with lower odds of insufficient and poor sleep compared to high performance (AOR=0.85; 95% CI: 0.82–0.88). Daily phone use ≥6 hours was linked to higher odds of insufficient and poor sleep (AOR=1.86; 95% CI: 1.77–1.97), and sedentary time ≥11 hours per day showed a strong association with poor sleep (AOR=2.53; 95% CI: 2.43–2.63).

### Association between tobacco use behavior and sleep outcomes

**Figure 3:**
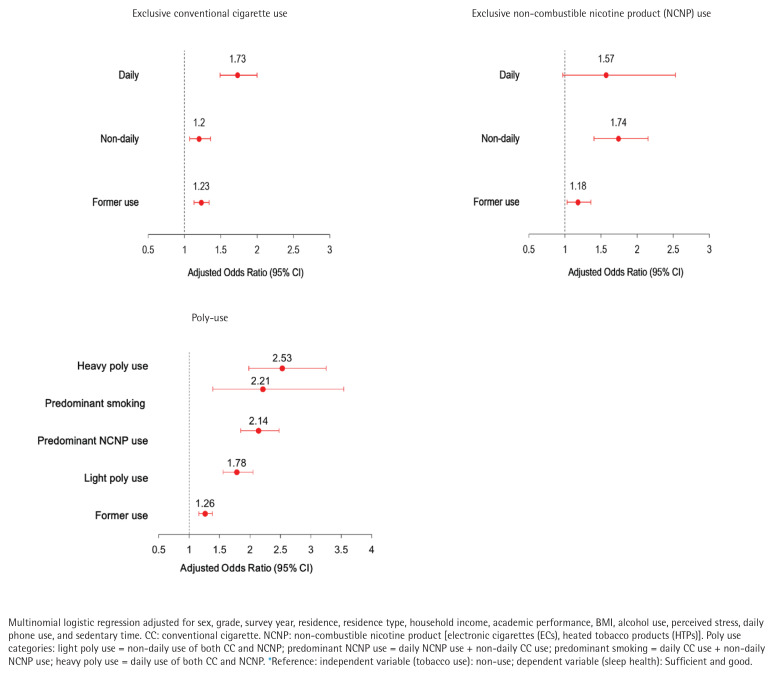
Forest plots of adjusted odds ratios (AOR, 95% CI) for insufficient and poor sleep, by tobacco use behavior vs reference*, South Korea, Korea Youth Risk Behavior Web-based Survey (KYRBS), 2020–2023

Table 4 presents associations by detailed tobacco use behavior. Adolescents who smoked conventional cigarettes daily had increased odds of insufficient and poor sleep compared to non-users (AOR=1.73; 95% CI: 1.49–2.00). Among NCNP users, daily users showed higher odds of insufficient and poor sleep (AOR=1.57; 95% CI: 0.97–2.53), though this was not statistically significant, while former NCNP users had an AOR of 1.18 (95% CI: 1.03–1.36). Patterns were more pronounced in poly users: predominant cigarette users (AOR=2.14; 95% CI: 1.85–2.48), predominant NCNP users (AOR=2.21; 95% CI: 1.39–3.54), and heavy poly users (AOR=2.53; 95% CI: 1.98–3.25) all showed markedly higher likelihood of insufficient and poor sleep compared with non-users (Figure 3). Exploratory interaction analyses suggested that the association between tobacco use and sleep health varied by alcohol use, stress, and grade level. Detailed results are presented in Supplementary file Appendix B.

**Table 4:** Multinomial logistic regression, including all covariates, for associations between tobacco use behavior and sleep health among adolescents, Korea Youth Risk Behavior Web-based Survey 2020–2023 (N=172457)

*Tobacco use behavior (ref: non-use)*		*Sleep health* *(ref: sufficient and good)*		
		*Insufficient and poor*	*Sufficient and poor*	*Insufficient and good*
		*AOR (95% CI)*	*AOR (95% CI)*	*AOR (95% CI)*
**Exclusive CC use**	Former use	1.23 (1.13–1.34) ***	1.17 (1.06–1.29) *	1.11 (1.02–1.21) *
	Non-daily	1.20 (1.07–1.36) *	1.02 (0.87–1.19)	1.03 (0.90–1.17)
	Daily	1.73 (1.49–2.00) ***	1.21 (0.99–1.47)	1.18 (1.01–1.38) *
**Exclusive NCNP use**	Former use	1.18 (1.03–1.36) *	1.16 (0.98–1.36)	1.11 (0.96–1.28)
	Non-daily	1.74 (1.40–2.15) ***	1.26 (0.97–1.64)	1.44 (1.14–1.81) *
	Daily	1.57 (0.97–2.53)	1.46 (0.81–2.61)	1.32 (0.80–2.20)
**Poly use^a^**	Former use	1.26 (1.16–1.38) ***	1.30 (1.17–1.44)	1.06 (0.96–1.16) ***
	Light poly use	1.78 (1.56–2.05) ***	1.32 (1.12–1.56) *	1.39 (1.20–1.62) ***
	Predominant NCNP use	2.14 (1.85–2.48) ***	1.53 (1.27–1.84) ***	1.48 (1.27–1.73) ***
	Predominant smoking	2.21 (1.39–3.54) **	2.06 (1.19–3.57) *	1.57 (0.94–2.63)
	Heavy poly use	2.53 (1.98–3.25) ***	1.47 (1.06–2.03) *	1.46 (1.11–1.93) *

Models adjusted for sex, grade level, survey year, residence, type of residence, household income, academic performance, body mass index, alcohol use, perceived stress, daily phone use, and sedentary time for academic purposes. AOR: adjusted odds ratio. CC: conventional cigarette. NCNP: non-combustible nicotine product, including electronic cigarettes (ECs) and heated tobacco products (HTPs).

a Poly use categories: light poly use = non-daily use of both CC and NCNP; predominant NCNP use = daily NCNP use + non-daily CC use; predominant smoking = daily CC use + non-daily NCNP use; and heavy poly use = daily use of both CC and NCNP.

* p<0.05;

** p<0.001;

*** p<0.0001.

## DISCUSSION

This study examined sleep duration and satisfaction among South Korean adolescents using nationally representative data from the KYRBS. The findings showed that the likelihood of insufficient and poor sleep increased with the complexity of tobacco use behaviors, ranging from exclusive conventional cigarette use to multiple product use. Adolescents engaging in triple use exhibited the greatest likelihood of insufficient and poor sleep, followed by dual users. These results align with previous studies reporting that dual users of cigarettes and e-cigarettes are more likely to experience inadequate sleep and longer sleep latency compared to non-users^23,24^. Nicotine is known to disrupt sleep architecture by prolonging sleep latency and reducing overall sleep quality, and such effects may be particularly pronounced during adolescence, a developmental stage characterized by heightened vulnerability^13,25^.

Importantly, the study also observed that as the frequency of poly use increased, the likelihood of insufficient and poor sleep rose progressively. This pattern is consistent with prior research showing that higher nicotine exposure, reflected in serum cotinine levels, is associated with poor sleep quality^26,27^. The findings underscore the cumulative effects of poly use and expand upon earlier studies that focused primarily on single-product use.

Sociocultural and educational pressures may also contribute to poor sleep among South Korean adolescents. Average sleep duration remains well below recommended levels^4,5^. This shortfall is driven by intensive academic schedules, prolonged sedentary time, increased screen exposure, and caffeine consumption, which may interact with tobacco use to exacerbate sleep problems^28-31^. Exploratory interaction analyses, reported in the Supplementary file, suggest that the impact of tobacco use on sleep health may be modified by behavioral and psychosocial factors such as alcohol use, stress, and academic stage. Future studies should confirm these patterns using longitudinal designs.

### Strengths and limitations

The study benefits from notable strengths. The use of a large, nationally representative sample of South Korean adolescents allows for generalizability within this population; thus, generalizability to other countries may be limited. The four-year span provides robust statistical power to detect associations. Furthermore, the inclusion of detailed tobacco use patterns, including dual and triple use, offers insights into how increasingly complex behaviors relate to adolescents’ sleep health.

This study has, however, several limitations. First, its cross-sectional design prevents causal inference, and the possibility of reverse causality cannot be excluded. For example, adolescents with poor sleep may be more likely to initiate or maintain tobacco use. Second, although covariates were carefully selected, the potential for residual confounding remains, as unmeasured factors such as dietary habits or mental health status may influence both tobacco use and sleep. Third, reliance on self-reported measures may introduce information bias and misclassification. Finally, categorization of tobacco use behaviors by days used in the past 30 days may not fully capture intensity or graded patterns.

## CONCLUSIONS

This study provides evidence that complex tobacco use behaviors, particularly dual and triple use, are associated with poorer sleep health among South Korean adolescents. By incorporating a large, nationally representative sample and distinguishing between different patterns of tobacco use, the findings contribute to a more nuanced understanding of how tobacco use behaviors relate to adolescent well-being. While the cross-sectional design limits causal inference, the results highlight important areas for future longitudinal research to better clarify temporal relationships and mechanisms. Recognizing these links in adolescence may be critical for informing future studies aimed at understanding the long-term health consequences of tobacco use.

## Supplementary Material



## Data Availability

The data supporting this research are available from the authors on reasonable request.
