# Supplementary material for: Association between smoking behavior and sleep health among South Korean adolescents: A cross-sectional study 2020–2023
Source: Tob Induc Dis. 2025 Dec 31;23:10.18332/tid/211247. doi: 10.18332/tid/211247 (PMC12754695; doi:10.18332/tid/211247)
Supplement: Supplementary file 1 [file TID-23-199-s1.pdf]

Supplementary Materials

Appendix A. Multinomial logistic regression of covariates with four-category sleep health among South Korean adolescents, KYRBS 2020–2023 (N = 172,457).

**Variables**		**Sleep Health (ref. Sufficient & Good)**		
		**Sufficient & Poor**	**Insufficient & Good**	**Insufficient & Poor**
		**AOR (95% CI)**	**AOR (95% CI)**	**AOR (95% CI)**
**Sex (ref: male)**	Female	1.28 (1.24-1.32) ***	1.49 (1.45-1.53) ***	2.09 (2.03-2.15) ***
**Grade level (ref: 7th)**	8th	1.10 (1.05-1.15) ***	1.32 (1.26-1.38) ***	1.67 (1.60-1.75) ***
	9th	1.20 (1.15-1.26) ***	1.73 (1.65-1.82) ***	2.34 (2.23-2.45) ***
	10th	1.24 (1.17-1.31) ***	3.39 (3.23-3.56) ***	5.16 (4.92-5.42) ***
	11th	1.15 (1.09-1.22) ***	3.98 (3.78-4.18) ***	5.41 (5.14-5.68) ***
	12th	1.02 (0.96-1.08)	3.90 (3.71-4.10) ***	5.00 (4.75-5.26) ***
**Type of residential area (ref: rural area)**	Small to mid-sized city	1.01 (0.95-1.06)	1.16 (1.10-1.22) ***	1.35 (1.28-1.43) ***
	Metropolitan city	0.95 (0.90-1.01)	1.42 (1.34-1.50) ***	1.66 (1.57-1.75) ***
**Household income level (ref: high)**	Moderate	1.13 (1.09-1.17) ***	0.96 (0.93-0.99) *	1.04 (1.01-1.07) *
	Low	1.39 (1.32-1.47) ***	0.95 (0.91-1.00) *	1.20 (1.15-1.25) ***
**Type of residence (ref: living with family)**	Dormitory	1.51 (1.38-1.65) ***	0.90 (0.82-0.98) *	1.11 (1.02-1.20) *
	Others	0.93 (0.80-1.08)	0.99 (0.87-1.14)	0.96 (0.84-1.09)
**Academic**	Moderate	0.89 (0.85-0.92)	1.11 (1.08-1.15)	0.85 (0.82-0.88)

**performance (ref: high)**		***	***	***
	Low	1.01 (0.97-1.05)	1.20 (1.15-1.24) ***	0.92 (0.89-0.95) ***
**BMI level[a] (ref: normal)**	Obese	0.87 (0.84-0.92) ***	1.01 (0.97-1.05)	0.86 (0.82-0.90) ***
	Overweight	0.94 (0.89-0.99) *	1.00 (0.96-1.05)	0.92 (0.88-0.97) **
	Underweight	0.98 (0.93-1.04)	0.98 (0.94-1.03)	0.96 (0.91-1.00)
**Alcohol use (ref: no)**	Yes	1.27 (1.23-1.32) ***	1.19 (1.15-1.23) ***	1.43 (1.39-1.48) ***
**Perceived stress (ref: below high)**	High	2.44 (2.37-2.52) ***	1.29 (1.25-1.33) ***	3.13 (3.04-3.21) ***
**Phone use per day (ref: less than 2 hours)**	More than 6 hours	1.26 (1.18-1.33) ***	1.77 (1.68-1.87) ***	1.86 (1.77-1.97) ***
	4-6 hours	1.21 (1.14-1.27) ***	1.41 (1.34-1.49) ***	1.42 (1.35-1.49) ***
	2-4 hours	1.11 (1.05-1.17) **	1.14 (1.09-1.20) ***	1.16 (1.11-1.22) ***
**Sedentary time (for academic purposes) (ref: <7 hours)**	7-11 hours	1.18 (1.14-1.22) ***	0.91 (0.88-0.93) ***	1.16 (1.13-1.20) ***
	≥11 hours	1.47 (1.40-1.54) ***	1.44 (1.38-1.51) ***	2.53 (2.43-2.63) ***
**Survey year (ref: 2020)**	2021	1.49 (1.43-1.56) ***	0.79 (0.76-0.82) ***	1.16 (1.12-1.20) ***
	2022	1.49 (1.42-1.55) ***	0.80 (0.77-0.84) ***	1.23 (1.19-1.28) ***
	2023	1.20 (1.15-1.26) ***	0.66 (0.64-0.69) ***	0.90 (0.86-0.93) ***

[i] Multinomial logistic regression with sleep health (ref: Sufficient & Good) as the dependent variable. All models accounted for KYRBS complex survey design (weights, strata, clusters) and adjusted for sex, grade level, survey year, residential area, type of residence, household income, academic performance, body mass index (BMI), alcohol use, perceived stress, daily phone use, and sedentary time for academic purposes. Reporting: Adjusted odds ratios (AOR) and 95% confidence intervals (CI). BMI = body mass index; CI = confidence interval; AOR = adjusted odds ratio; ref. = reference category; p = p-value; KYRBS = Korea Youth Risk Behavior Web-based Survey.

[a]BMI level: underweight (<18.5 kg/m²), normal (18.5–24.9 kg/m²), overweight (25.0–29.9 kg/m²), obese (≥30.0 kg/m²)*p<0.05; **p<0.001; ***p<0.0001

Appendix B. Interaction analyses of tobacco use and sleep health (binomial models), KYRBS 2020–2023 (N = 172,457).

Variables	Estimate	Standard Error	p-value	Exp (Est)
Intercept	-1.733	0.025	<.0001	0.177
Tobacco use	0.702	0.054	<.0001	2.017
Sex	0.508	0.011	<.0001	1.662
Grade level	1.123	0.011	<.0001	3.075
Type of residential area	0.342	0.021	<.0001	1.407
Household income level	-0.017	0.011	0.137	0.983
Type of residence	-0.066	0.028	0.018	0.936
Academic performance	0.037	0.012	0.002	1.038
BMI level[a]	-0.034	0.013	0.008	0.967
Alcohol use	0.228	0.012	<.0001	1.256
Perceived stress	0.480	0.011	<.0001	1.617
Phone use per day	0.318	0.011	<.0001	1.374
Sedentary time (for academic purposes)	0.239	0.011	<.0001	1.269
Survey year	-0.147	0.011	<.0001	0.864
Tobacco use*grade level	-0.387	0.061	<.0001	0.679
Tobacco use*sedentary time	-0.128	0.061	0.035	0.880

[i] Binomial logistic regression with sleep health dichotomized as Poor vs. Good to improve interpretability and subgroup cell sizes. Interaction terms tested modification by grade level and sedentary time (and other specified moderators). Full model covariate set matches Appendix A; complex survey design was incorporated.

Estimate is the log-odds coefficient; Exp(Est) is the odds ratio; SE = standard error; p = p-value; BMI = body mass index; KYRBS = Korea Youth Risk Behavior Web-based Survey.

[a]BMI level: underweight (<18.5 kg/m²), normal (18.5–24.9 kg/m²), overweight (25.0–29.9 kg/m²), obese (≥30.0 kg/m²).

© 2025 Cho N. et al.
